# A Distinct Defense Strategy: The Molecular Basis of WSSV Tolerance in *Macrobrachium nipponense* Revealed by Comparative Transcriptomics with *Litopenaeus vannamei*

**DOI:** 10.3390/ijms27020766

**Published:** 2026-01-12

**Authors:** Yunpeng Niu, Sufei Jiang, Wenyi Zhang, Yiwei Xiong, Shubo Jin, Hui Qiao, Hongtuo Fu

**Affiliations:** 1 Wuxi Fisheries College, Nanjing Agricultural University, Wuxi 214081, China; 2 Key Laboratory of Freshwater Fisheries and Germplasm Resources Utilization, Ministry of Agriculture and Rural Affairs, Freshwater Fisheries Research Center, Chinese Academy of Fishery Sciences, Wuxi 214081, China

**Keywords:** *Macrobrachium nipponense*, *Litopenaeus vannamei*, white spot syndrome virus (WSSV), comparative transcriptomics

## Abstract

White Spot Syndrome Virus (WSSV) remains one of the most devastating pathogens in global shrimp aquaculture, causing massive economic losses annually. This study employed comparative transcriptomics to elucidate the molecular basis of the differential resistance to WSSV between the highly susceptible Pacific white shrimp (*Litopenaeus vannamei*) and the remarkably resistant oriental river prawn (*Macrobrachium nipponense*). Our analysis of gill, hepatopancreas, and muscle tissues at 24 h post-infection revealed fundamentally distinct defense strategies. The resistant *M. nipponense* employs a unique “proactive homeostatic reinforcement” strategy, characterized by significant enrichment of pathways central to cellular homeostasis, including signal transduction, cellular processes, and transport/catabolism. This approach, supported by coordinated up-regulation of heat shock proteins and structural genes, enables effective viral control without triggering excessive immune activation. In contrast, susceptible *L. vannamei* displays either widespread metabolic dysregulation leading to systemic collapse in moribund individuals or dependency on specific immune pathways (Toll-like receptor signaling and apoptosis) in survivors. Through comparative KEGG analysis, we identified heat shock protein 70 kDa (*HSP70*, K03283) as a key conserved gene and functionally validated its critical role in antiviral defense using RNA interference. Knockdown of *HSP70* in *M. nipponense* significantly increased cumulative mortality and viral load, confirming its essential protective function. These findings provide novel insights into crustacean antiviral immunity and identify promising genetic targets for breeding WSSV-resistant shrimp strains, offering sustainable solutions for disease management in aquaculture.

## 1. Introduction

White Spot Syndrome Virus (WSSV) is recognized as one of the most economically devastating pathogens in global shrimp aquaculture [1]. It belongs to the family *Nimaviridae* and the genus Whispovirus, of which it is the sole member. WSSV is an enveloped, double-stranded DNA virus with a genome size of approximately 300 kb [2]. It exhibits a broad host range, capable of infecting a wide variety of crustaceans, including shrimp and crabs. The Pacific white shrimp, *Litopenaeus vannamei*, has become the most extensively farmed crustacean worldwide due to its high yield and broad salinity tolerance [3]. Over the past three decades, viral diseases have posed a severe threat to the global shrimp aquaculture industry [4,5]. Among these, White Spot Syndrome Virus (WSSV) stands out as a major viral pathogen, causing high mortality rates and substantial economic losses [6]. In most infected shrimp, early-stage infection manifests as lethargy, reduced feeding, anorexia, and decreased activity. As the infection progresses, typical signs include an easily removable cuticle, reddening of the body, and the appearance of white spots on the exoskeleton. Mortality rates can reach 100% within 3 to 10 days post-infection [6,7,8]. To date, no effective therapeutic treatment for this disease has been developed. Furthermore, WSSV transmission occurs through both vertical and horizontal routes, complicating disease management and control efforts [9]. These characteristics collectively make the prevention and control of WSSV extremely challenging. Consequently, understanding shrimp immune responses to viral pathogens has become an increasingly critical research focus. Elucidating the molecular mechanisms underlying host defense and pathogen interactions in shrimp is essential for developing effective strategies to combat viral diseases in aquaculture.

The oriental river prawn, *Macrobrachium nipponense*, is an economically important freshwater species widely cultivated in China. It predominantly inhabits rivers and lakes, and is characterized by its strong adaptability, high fecundity, and rapid growth rate [10]. Notably, during cultivation, *M. nipponense* has been observed to carry WSSV without developing clinical signs of disease, indicating a greater resistance to WSSV compared to *L. vannamei.* Previous studies have demonstrated that the median lethal dose (LD_50_) of WSSV in *M. nipponense* is 10^3.84±0.06^ copies/g under identical experimental conditions, which is over 1000 times higher than that in *L. vannamei* (10^0.59±0.22^ copies/g) [3,11]. Furthermore, our current investigation confirms that WSSV can infect multiple tissues in *M. nipponense*, including the gills, muscle, stomach, hepatopancreas, heart, intestine, ventral nerve, epidermis, pereiopods, eyestalk, testis, and ovary. Among these, the viral load was notably higher in the gills, hepatopancreas, and muscle than in other tissues.

In recent years, the application of next-generation high-throughput sequencing technologies has driven breakthroughs across various biological fields, including animal genomics [4,12,13]. Transcriptomics has been widely employed in shrimp research, serving as a powerful tool for identifying genes and proteins involved in immune and metabolic pathways. Among these technologies [14,15,16], Illumina RNA-Seq is particularly suited for transcriptomic studies of non-model species like *M. nipponense*, owing to its high data output, rapid turnaround, cost-effectiveness, and robust performance.

In this study, we employed a comparative transcriptomics approach to investigate the molecular mechanisms underlying the response to White Spot Syndrome Virus (WSSV) in *M. nipponense* and *L. vannamei*. Using high-throughput sequencing, we conducted a comparative transcriptomic analysis of three tissues—gills, hepatopancreas, and muscle—from both species at 24 h post-WSSV infection. This analysis aimed to identify common candidate genes involved in WSSV resistance shared between *L. vannamei* and *M. nipponense*. Furthermore, the function of the candidate antiviral genes identified in *M. nipponense* was validated using RNA interference (RNAi) technology. Our work provides valuable insights and a genetic resource for understanding WSSV resistance mechanisms in shrimp.

## 2. Results

### 2.1. Overview of the Transcriptomes of M. nipponense and L. vannamei

RNA sequencing was performed on samples of *M. nipponense* and *L. vannamei*, generating high-quality data. A total of 18 and 27 transcriptomic libraries were constructed for *M. nipponense* and *L. vannamei*, respectively. For *M. nipponense*, the average number of reads per sample was 41,043,717, yielding approximately 6.16 billion base pairs of raw data. After filtering, an average of 40,813,214 high-quality reads (approximately 6.05 billion bases) were retained per sample. The average Q20 and Q30 scores were 98.40% and 95.21%, respectively, with a mean GC content of 45.04% and an average mapping rate of 88.57% to the reference genome. Supplementary Table S1 provides detailed sequencing statistics and quality control indicators for each bank of *M. nipponense*. For *L. vannamei*, each sample produced an average of 42,814,872 raw reads (approximately 6.42 billion bases). After quality control, an average of 42,549,527 clean reads (approximately 6.30 billion bases) were obtained. The average Q20 and Q30 values were 98.15% and 94.54%, respectively, with a mean GC content of 47.45% and an average mapping rate of 88.83%. Supplementary Table S2 provides detailed sequencing statistics and quality control indicators for each bank of *L. vannamei*. All sequence reads were deposited in NCBI (accession SRX31176670–SRX31176714) under Bioproject PRJNA1367779.

### 2.2. Screening of Differentially Expressed Genes

Comparative transcriptomic analysis was performed on three comparison sets in *M. nipponense* (M-Co-Gi vs. M-Su-Gi, M-Co-He vs. M-Su-He, M-Co-Mu vs. M-Su-Mu) and six comparison sets in *L. vannamei* (L-Co-Gi vs. L-Mo-Gi, L-Co-Gi vs. L-Su-Gi, L-Co-He vs. L-Mo-He, L-Co-He vs. L-Su-He, L-Co-Mu vs. L-Mo-Mu, L-Co-Mu vs. L-Su-Mu) to identify differentially expressed genes (DEGs) involved in the response to WSSV infection in gills, hepatopancreas, and muscle tissues of both species. Abbreviations: M, *M. nipponense*; L, *L. vannamei*; Co, control group; Mo, moribund group; Su, surviving group; Gi, gills; He, hepatopancreas; Mu, muscle.

In *M. nipponense*, 91 DEGs were identified in the M-Co-Gi vs. M-Su-Gi comparison, comprising 64 up-regulated and 27 down-regulated genes. The M-Co-He vs. M-Su-He comparison revealed 13 DEGs, with 12 being up-regulated and 1 down-regulated. Furthermore, 133 DEGs were found in the M-Co-Mu vs. M-Su-Mu comparison, of which 12 were up-regulated and 121 were down-regulated.

In *L. vannamei*, transcriptomic analysis of gill tissue identified 824 differentially expressed genes (425 up-regulated and 399 down-regulated) in the L-Co-Gi vs. L-Mo-Gi comparison, and 34 DEGs (11 up-regulated and 23 down-regulated) in the L-Co-Gi vs. L-Su-Gi comparison. In the hepatopancreas, 1299 DEGs (623 up-regulated and 676 down-regulated) were detected in the L-Co-He vs. L-Mo-He comparison, while 105 DEGs (32 up-regulated and 73 down-regulated) were found in the L-Co-He vs. L-Su-He comparison. Regarding muscle tissue, the L-Co-Mu vs. L-Mo-Mu comparison revealed 338 DEGs (257 up-regulated and 81 down-regulated), and the L-Co-Mu vs. L-Su-Mu comparison showed 10 DEGs (4 up-regulated and 6 down-regulated).

Analysis of DEGs in the survival group versus the control group of *M. nipponense* revealed that the down-regulated genes were primarily associated with cuticle proteins, exoskeletal proteins, and chitin metabolic enzymes. In contrast, the up-regulated genes were dominated by actin family members, heat shock proteins (e.g., *HSP21*, *HSP70*), and apolipoprotein D-like proteins. These transcriptional alterations suggest a coordinated physiological response in surviving *M. nipponense*, characterized by reduced exoskeletal metabolism, enhanced muscle structure, and elevated expression of stress-related proteins.

In the moribund group versus control group of *L. vannamei*, down-regulated genes were predominantly enriched for cuticle proteins (e.g., cuticle protein AMP1A-like, CP14.1), immune-related proteins (such as GPI inositol deacylase, chitinase, and C-type lectins), and structural proteins (including actin and myosin heavy chain). Conversely, the up-regulated genes were chiefly composed of heat shock protein family members (*HSP21*, *HSP70*), stress response proteins (e.g., hemocyanin, thrombospondin), and metabolism-associated proteins (such as glucose dehydrogenase). This expression profile indicates a state of severe pathophysiological stress in moribund *L. vannamei*, marked by apparent immunosuppression, degradation of structural proteins, and a concurrent strong up-regulation of stress response genes.

**Figure 1:**
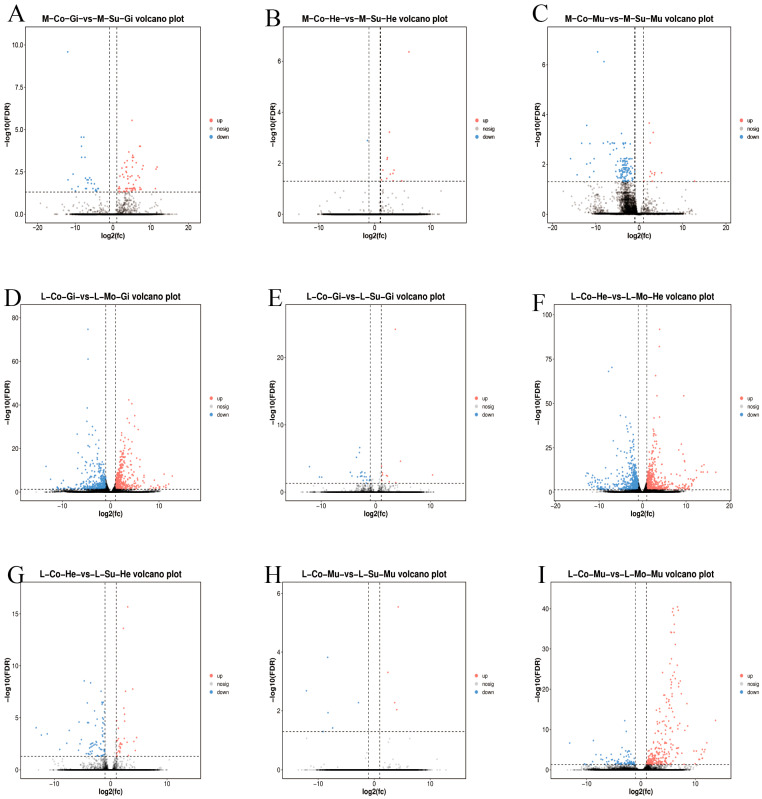
Volcano plots of differentially expressed genes (DEGs) in *M. nipponense* and *L. vannamei* under survival stress across different tissues. The nine panels display DEGs from the following pairwise comparisons between the control (Co), moribund (Mo), and surviving (Su) groups: (**A**) M-Co-Gi vs. M-Su-Gi (gills), (**B**) M-Co-He vs. M-Su-He (hepatopancreas), and (**C**) M-Co-Mu vs. M-Su-Mu (muscle), (**D**) L-Co-Gi vs. L-Mo-Gi (gills), (**E**) L-Co-Gi vs. L-Su-Gi (gills), (**F**) L-Co-He vs. L-Mo-He (hepatopancreas), (**G**) L-Co-He vs. L-Su-He (hepatopancreas). (**H**) L-Co-Mu vs. L-Su-Mu (muscle), (**I**) L-Co-Mu vs. L-Mo-Mu (muscle). For all plots, the x-axis represents the log2 fold change (log2FC), and the y-axis represents the statistical significance as −log10 of the false discovery rate (FDR). Genes with significant up-regulation and down-regulation are highlighted in red and blue, respectively, based on thresholds of |log_2_FC| > 1 and FDR < 0.05. Horizontal dashed line indicates the significance threshold of FDR < 0.05. Vertical dashed lines correspond to the fold change thresholds of |log_2_FC| > log_2_(2). Abbreviations: M, *M. nipponense*; L, *L. vannamei*; Co, Control group; Mo, Moribund group; Su, Surviving group; Gi, Gills; He, Hepatopancreas; Mu, Muscle.

Regarding the survival group versus control group of *L. vannamei*, only a limited number of genes were down-regulated, including troponin and caspase-3, which are implicated in apoptosis and muscle function. The up-regulated genes were primarily associated with immune enhancement, such as hemocyanin, lectins, and peroxidases, alongside a moderate up-regulation of actin and myosin. This pattern suggests that surviving *L. vannamei* mounted an effective immune response while maintaining muscle function. Figure 1 shows the volcano plots of differentially expressed genes from the nine comparison sets: M-Co-Gi vs. M-Su-Gi, M-Co-He vs. M-Su-He, M-Co-Mu vs. M-Su-Mu, L-Co-Gi vs. L-Mo-Gi, L-Co-Gi vs. L-Su-Gi, L-Co-He vs. L-Mo-He, L-Co-He vs. L-Su-He, L-Co-Mu vs. L-Su-Mu, and L-Co-Mu vs. L-Mo-Mu.

### 2.3. GO and KEGG Enrichment Analysis of Differentially Expressed Genes

This study identified DEGs from *M. nipponense* and *L. vannamei* under WSSV challenge through the aforementioned analyses. GO and KEGG analyses were subsequently employed to decipher the disparities in molecular regulation between the two species in response to WSSV infection.

GO enrichment analysis assigned DEGs from each comparison set to three primary categories: Biological Process, Molecular Function, and Cellular Component. In *M. nipponense*, the M-Co-Gi vs. M-Su-Gi comparison revealed enrichment for 21 Biological Process, 10 Molecular Function, and 3 Cellular Component terms. Among these, the “cellular anatomical entity” term was the most significantly enriched, containing 40 DEGs. For the M-Co-He vs. M-Su-He comparison, DEGs were enriched in 15 Biological Process, 5 Molecular Function, and 2 Cellular Component terms, with “cellular anatomical entity” again being the most prominent term (9 DEGs). In the M-Co-Mu vs. M-Su-Mu comparison, enrichment was observed for 23 Biological Process, 13 Molecular Function, and 3 Cellular Component terms, where the “cellular process” term contained the highest number of DEGs (60 genes).

**Figure 2:**
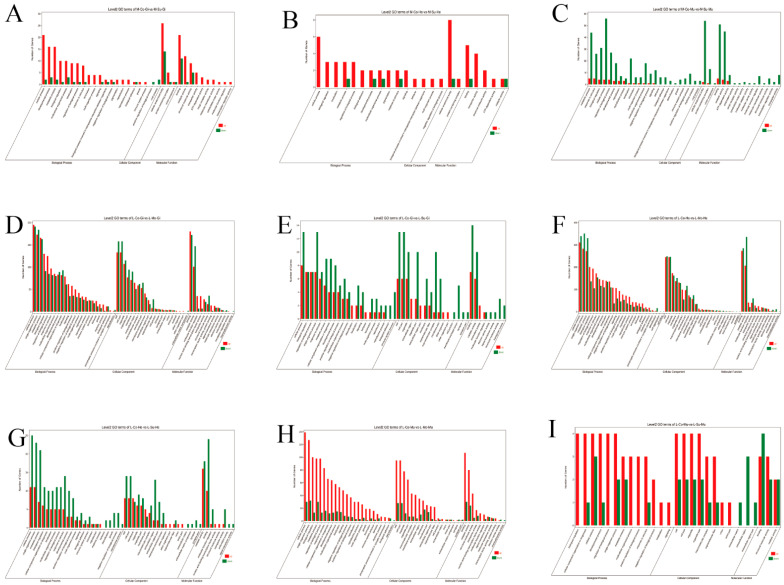
Gene Ontology (GO) enrichment analysis of differentially expressed genes in three tissues of *M. nipponense* and *L. vannamei* under WSSV challenge. (**A**–**I**) Bar graphs display the significantly enriched GO terms (Biological Process) for the comparative groups: (**A**) M-Co-Gi vs. M-Su-Gi (gills), (**B**) M-Co-He vs. M-Su-He (hepatopancreas), and (**C**) M-Co-Mu vs. M-Su-Mu (muscle), (**D**) L-Co-Gi vs. L-Mo-Gi (gills), (**E**) L-Co-Gi vs. L-Su-Gi (gills), (**F**) L-Co-He vs. L-Mo-He (hepatopancreas), (**G**) L-Co-He vs. L-Su-He (hepatopancreas). (**H**) L-Co-Mu vs. L-Mo-Mu (muscle), (**I**) L-Co-Mu vs. L-Su-Mu (muscle). The y-axis lists the enriched GO terms. The x-axis represents the number of differentially expressed genes (DEGs). Each bar is color-coded to represent the regulation direction of the DEGs: red for up-regulated and green for down-regulated in the Su (or Mo) group compared to the Co group. Data are derived from RNA-seq analysis.

In *L. vannamei*, the L-Co-Gi vs. L-Mo-Gi comparison showed enrichment for 24 Biological Process, 13 Molecular Function, and 21 Cellular Component terms, with the “cellular process” term containing the highest number of DEGs (386 genes). The L-Co-Gi vs. L-Su-Gi comparison revealed enrichment in 21 Biological Process, 8 Molecular Function, and 16 Cellular Component terms, where both “cellular process” and “binding” terms were most significantly enriched (21 DEGs each). For the L-Co-He vs. L-Mo-He comparison, DEGs were enriched in 26 Biological Process, 12 Molecular Function, and 23 Cellular Component terms, with “cellular process” being the most prominent category (650 DEGs). In the L-Co-He vs. L-Su-He comparison, enrichment was observed for 23 Biological Process, 8 Molecular Function, and 18 Cellular Component terms, and the “cellular process” term again contained the largest number of DEGs (36 genes). Regarding the muscle tissue comparisons, the L-Co-Mu vs. L-Mo-Mu set showed enrichment for 24 Biological Process, 11 Molecular Function, and 18 Cellular Component terms, with “cellular process” being the most enriched (169 DEGs). Finally, the L-Co-Mu vs. L-Su-Mu comparison demonstrated enrichment in 25 Biological Process, 12 Molecular Function, and 20 Cellular Component terms, where the “cellular process” category contained the highest number of DEGs (260 genes). Across all comparison sets in both species, the Biological Process category contained the largest number of enriched functional terms (q-value < 0.05; Figure 2).

**Figure 3:**
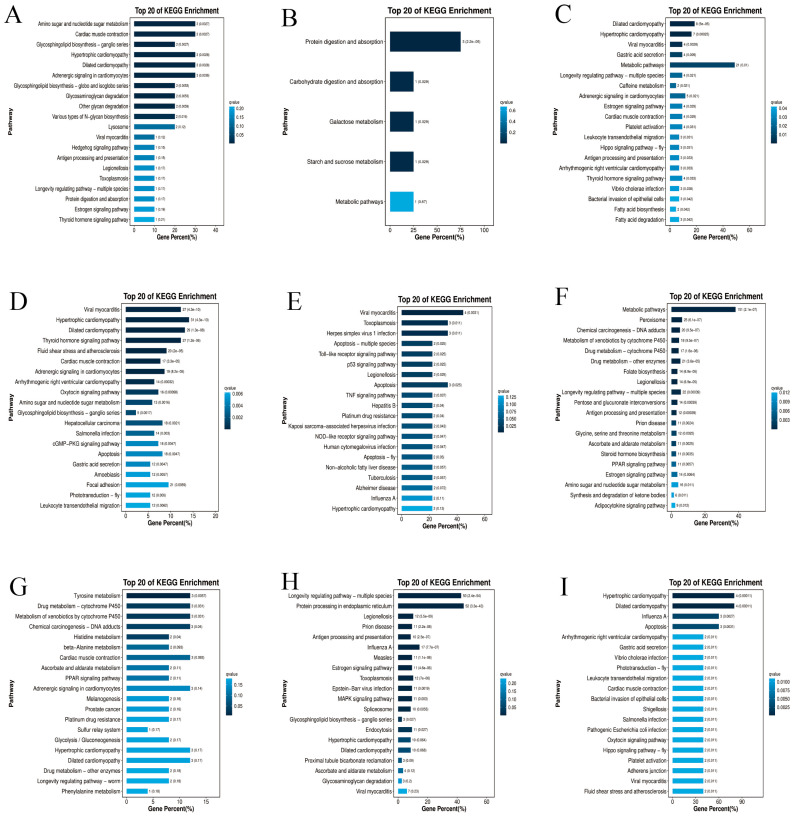
Kyoto Encyclopedia of Genes and Genomes (KEGG) pathway enrichment analysis of the top 20 significantly enriched terms for differentially expressed genes (DEGs) between *M. nipponense* and *L. vannamei*. Bar charts display the results from three tissue-specific comparative groups: (**A**) M-Co-Gi vs. M-Su-Gi (gills), (**B**) M-Co-He vs. M-Su-He (hepatopancreas), and (**C**) M-Co-Mu vs. M-Su-Mu (muscle), (**D**) L-Co-Gi vs. L-Mo-Gi (gills), (**E**) L-Co-Gi vs. L-Su-Gi (gills), (**F**) L-Co-He vs. L-Mo-He (hepatopancreas), (**G**) L-Co-He vs. L-Su-He (hepatopancreas). (**H**) L-Co-Mu vs. L-Mo-Mu (muscle), (**I**) L-Co-Mu vs. L-Su-Mu (muscle). The y-axis lists the top 20 enriched KEGG pathways. The x-axis represents the gene percentage (%), indicating the proportion of DEGs mapped to a specific pathway relative to the total genes in that pathway. The color gradient of the bars corresponds to the enrichment significance, represented by the qvalue (a corrected *p*-value), with a more intense color indicating a more significant enrichment. Numbers on the x-axis represent gene counts and are displayed in scientific notation.

Concurrently, KEGG pathway enrichment analysis was performed on all DEGs from each comparison group. In *M. nipponense*, 91 DEGs from the M-Co-Gi vs. M-Su-Gi comparison were mapped to 30 KEGG pathways; 13 DEGs from the M-Co-He vs. M-Su-He comparison were enriched in 5 pathways; and 133 DEGs from the M-Co-Mu vs. M-Su-Mu comparison were assigned to 140 pathways. In *L. vannamei*, 824 DEGs (L-Co-Gi vs. L-Mo-Gi) were enriched in 270 pathways; 34 DEGs (L-Co-Gi vs. L-Su-Gi) were mapped to 81 pathways; 1299 DEGs (L-Co-He vs. L-Mo-He) were associated with 288 pathways; 105 DEGs (L-Co-He vs. L-Su-He) were assigned to 57 pathways; 338 DEGs (L-Co-Mu vs. L-Mo-Mu) were enriched in 191 pathways; and 10 DEGs (L-Co-Mu vs. L-Su-Mu) were mapped to 39 pathways. The top 20 most significantly enriched KEGG pathways from all comparison groups are presented in Figure 3.

In *M. nipponense*, transcriptomic analysis of gill tissue from the survival group versus the control group revealed significant enrichment of differentially expressed genes in pathways including amino sugar and nucleotide sugar metabolism, cardiac muscle contraction, glycosphingolipid biosynthesis—ganglio series, hypertrophic cardiomyopathy, and dilated cardiomyopathy. In the hepatopancreas, DEGs were predominantly enriched in pathways related to protein digestion and absorption, carbohydrate digestion and absorption, and galactose metabolism. Meanwhile, in muscle tissue, the DEGs showed significant enrichment in pathways associated with dilated cardiomyopathy, hypertrophic cardiomyopathy, and viral myocarditis.

In *L. vannamei*, distinct pathway enrichment patterns were observed between moribund and survival groups across different tissues. In gill tissue, DEGs from the moribund vs. control comparison were predominantly enriched in pathways including viral myocarditis, hypertrophic cardiomyopathy, and dilated cardiomyopathy. In contrast, DEGs from the survival vs. control comparison in gills showed significant enrichment in the Toll-like receptor signaling pathway, NOD-like receptor signaling pathway, and apoptosis. In hepatopancreas tissue, the moribund vs. control comparison revealed primary enrichment in metabolic pathways, metabolism of xenobiotics by cytochrome P450, and chemical carcinogenesis. Conversely, DEGs from the survival vs. control comparison in the hepatopancreas were mainly enriched in tyrosine metabolism and the PPAR signaling pathway. Regarding muscle tissue, the moribund vs. control comparison displayed significant enrichment in the longevity regulating pathway, protein processing in endoplasmic reticulum, and influenza A. Meanwhile, DEGs from the survival vs. control comparison in muscle were primarily enriched in specific infection pathways (Vibrio cholerae infection, Shigellosis), as well as cardiac muscle contraction.

### 2.4. Validation of Differentially Expressed Genes

**Figure 4:**
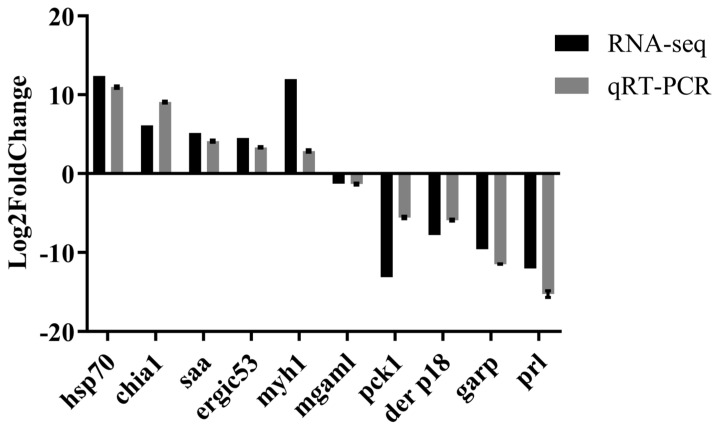
Validation of RNA−seq data by quantitative real-time PCR (qRT−PCR). The expression patterns of ten selected genes were analyzed to verify the reliability of the transcriptome sequencing data. Bar chart displays the fold-change in expression of the candidate genes as determined by RNA-seq and qRT−PCR across the compared groups. The genes validated include heat shock protein 70 kDa (*hsp70*), endochitinase A1−like (*chia1*), serum amyloid A (*saa*), ERGIC−53−like isoform X3 (*ergic53*), myosin heavy chain, muscle-like isoform X1 (*myh1*), maltase-glucoamylase, intestinal-like (*mgaml*), phosphoenolpyruvate carboxykinase, cytosolic [GTP]−like (*pckl*), chitinase-like mite allergen Der p 18.0101 (*der p18*), glutamic acid-rich protein−like (*garp*) and pro−resilin−like (*prl*). The consistent expression trends between the two methods confirm the accuracy of the RNA-seq results. The fold-change in gene expression for RNA-seq and qRT−PCR was determined using FPKM and the 2^−ΔΔCT^ method, respectively. Data are presented as mean ± SD from three independent biological replicates.

Ten DEGs were randomly selected for experimental validation by quantitative RT-PCR (qRT-PCR). These genes included heat shock protein 70 kDa (*hsp70*), endochitinase A1-like (*chia1*), serum amyloid A (*saa*), ERGIC-53-like isoform X3 (*ergic53*), myosin heavy chain, muscle-like isoform X1 (*myh1*), maltase-glucoamylase, intestinal-like (*mgaml*), phosphoenolpyruvate carboxykinase, cytosolic [GTP]-like (*pckl*), chitinase-like mite allergen Der p 18.0101 (*der p18*), glutamic acid-rich protein-like (*garp*) and pro-resilin-like (*prl*). The qRT-PCR results confirmed expression trends consistent with the transcriptome sequencing data, confirming the accuracy of transcriptome sequencing (Figure 4).

### 2.5. Screening of Common Candidate Genes

By comparing the KEGG pathway identifiers of the top 20 up- and down-regulated differentially expressed genes from each comparison group in *M. nipponense* and *L. vannamei*, we identified genes that exhibited consistent expression trends and shared the same KEGG ID in both species. Through this analysis, heat shock protein 70 kDa (Genbank accession No.: PX754291. KEGG ID: K03283) was selected as a common candidate gene for WSSV resistance in both *M. nipponense* and *L. vannamei*. Supplementary Table S3 provides a detailed list of the top 20 up- and down-regulated differentially expressed genes (DEGs) ranked by Log2FC for each comparison set.

### 2.6. Functional Validation of the Candidate Gene

(1) RNA Interference Efficiency

**Figure 5:**
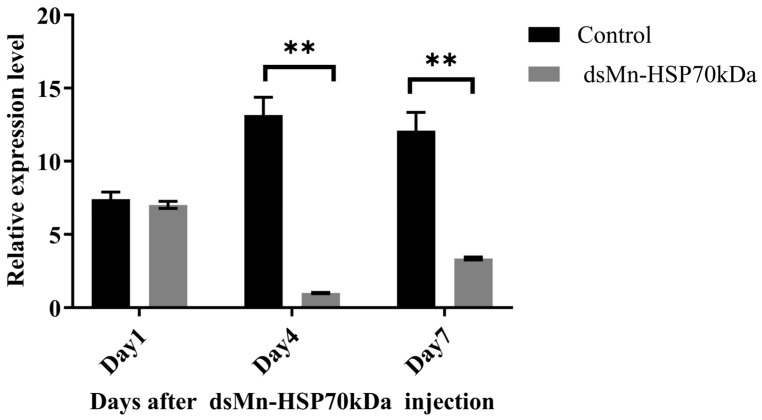
Expression levels in muscle after dsRNA injection (three biological replicates, three prawns/replicate). Data are presented as the mean ± standard. ** indicates highly significant difference (*p* < 0.01).

The short-term RNAi efficiency was monitored at 1, 4, and 7 days post-injection. The results demonstrated that dsRNA injection exhibited no significant effect on Mn-HSP70 expression at day 1 post-injection, with no statistically significant difference observed between the experimental and control groups (*p* > 0.05). In contrast, by day 4 post-injection, the expression level of Mn-HSP70 in the dsHSP70-treated group was significantly reduced by 96.61% compared to the control group (*p* < 0.01). A substantial knockdown effect persisted at day 7, with a 61.80% reduction in expression (*p* < 0.01), as illustrated in the accompanying Figure 5.

(2) WSSV Challenge Experiment in dsRNA-Treated *M. nipponense*

**Figure 6:**
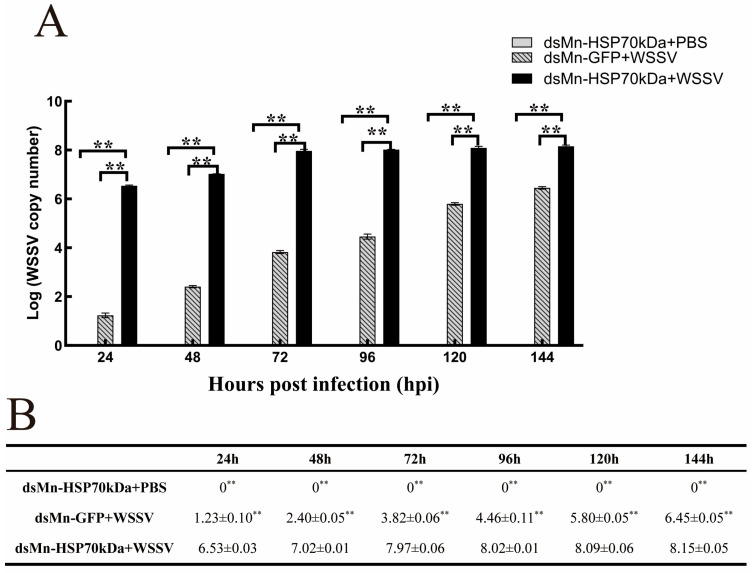
Effect of dsMn-HSP70kDa on WSSV copy number in the muscle tissue of *M. nipponense* challenged with WSSV. (**A**) Bar chart of WSSV copy number for the three groups at various time points. (**B**) WSSV copy number data for the three groups at various time points. The WSSV copy number was analyzed by qPCR at 24, 48, 72, 96, 120, and 144 h. Data are presented as the mean ± standard deviation. The data at each time point in the figure are mean ± SD, n = 9. ** indicates highly significant difference (*p* < 0.01).

Muscle tissues were collected from three experimental groups—dsMn-HSP70kDa + PBS, dsMn-GFP + WSSV, and dsMn-HSP70kDa + WSSV—at various time points from 0 to 144 h, and WSSV copy numbers were assessed by qRT-PCR. The results showed that no WSSV was detected in any muscle samples from the dsMn-HSP70kDa + PBS control group throughout the 0–144 h period. In contrast, the dsMn-HSP70kDa + WSSV experimental group exhibited significantly higher WSSV copy numbers at 24, 48, 72, 96, 120, and 144 h compared to the dsMn-GFP + WSSV control group. These findings indicate that injection of dsMn-HSP70kDa reduces the resistance of *M. nipponense* to WSSV infection, as evidenced by the increased viral load (Figure 6).

(3) Cumulative Mortality in dsRNA-Injected *M. nipponense* After WSSV Challenge

*M. nipponense* prawns were injected in the pericardial cavity with either dsMn-HSP70kDa or dsMn-GFP. On day 4 post-injection, which was determined from the interference efficiency experiment as the time of lowest target gene expression, the prawns were subsequently challenged with an injection of either WSSV viral suspension or PBS. The challenge dose was standardized to 10^3.58^ copies per gram of body weight with an injection volume of 2 µL. Mortality was recorded at 12 h intervals up to 144 h (12 h, 24 h, 36 h, 48 h, 60 h, 72 h, 84 h, 96 h, 108 h, 120 h, 132 h, 144 h).

The control group (ds-HSP70kDa + PBS) exhibited no mortality throughout the 0–144 h observation period. In the positive control group (dsMn-GFP + WSSV), the cumulative mortality increased progressively during the initial 0–120 h, remained stable from 120 h to 144 h, and ultimately reached a final mortality rate of 45.56% at 144 h.

**Figure 7:**
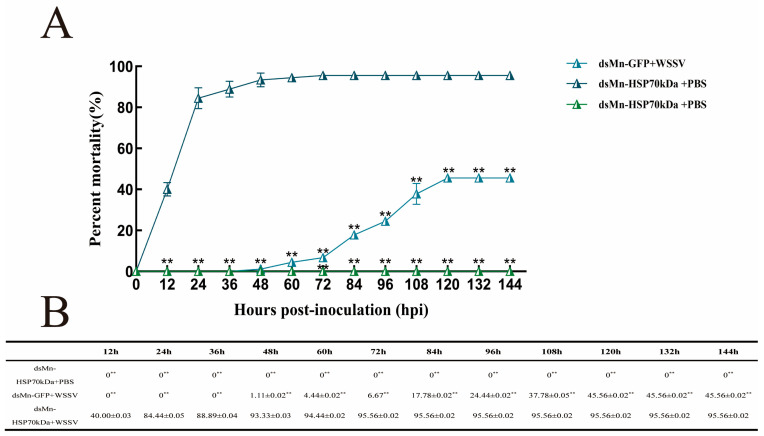
Cumulative mortality of *M. nipponense* challenged with WSSV from 0 to 144 h. (**A**) Line graph of cumulative mortality for the three groups at various time points. (**B**) Cumulative mortality data for the three groups at various time points. Shrimp in the experimental group were injected with dsMn-HSP70kDa and WSSV viral suspension. Control groups included shrimp injected with dsMn-HSP70kDa and PBS, and a positive control group injected with dsMn-GFP and WSSV. Timing began post-injection. Data represent the mean from three independent replicates. The data at each time point in the figure are mean ± SD, n = 9. ** indicates highly significant difference (*p* < 0.01).

In contrast, the experimental group (dsMn-HSP70kDa + WSSV) exhibited a progressive increase in cumulative mortality during the initial 0–72 h, maintained stable mortality levels from 72 h to 144 h, and ultimately reached a final cumulative mortality rate of 95.56% at 144 h. These data demonstrate that compared with the dsMn-GFP + WSSV control group, the dsMn-HSP70kDa + WSSV experimental group substantially increased the cumulative mortality of *M. nipponense* upon WSSV infection (Figure 7).

## 3. Discussion

According to statistics, approximately 60% of disease-related losses in shrimp aquaculture are attributed to viral pathogens, while 20% are caused by bacterial pathogens [17]. Currently, the list of critical viral pathogens affecting *L. vannamei* in Asia includes White Spot Syndrome Virus (WSSV), Yellow Head Virus genotype 1 (YHV1), and Infectious Myonecrosis Virus (IMNV) [18]. Among these, WSSV represents the most severe threat to all shrimp farming nations and cultured penaeid species in Asia. Both *L. vannamei* and *M. nipponense* are widely farmed crustaceans in China. However, *M. nipponense* exhibits significantly stronger tolerance to WSSV compared to *L. vannamei*. Investigating the response mechanisms underlying WSSV resistance in *M. nipponense* is therefore crucial for developing strategies to mitigate viral threats in other farmed crustaceans [3]. Consequently, we conducted a comparative transcriptomic analysis of gills, hepatopancreas, and muscle tissues from *M. nipponense* and *L. vannamei* at the same time point post-WSSV infection.

Our transcriptomic analysis revealed marked differences in the magnitude and nature of the transcriptional response between the two species. In *L. vannamei*, WSSV infection induced extensive transcriptomic alterations, with a total of 2610 differentially expressed genes (DEGs) identified across the three tissues. In contrast, *M. nipponense* exhibited a more modulated response, with only 237 DEGs detected in its survival group. As we know, WSSV can trigger a series of immediate-early (IE) and early genes to encode proteins involved in activating the expression of viral early and late genes, altering the functions of host genes and eliminating host immune defense [19,20,21,22,23,24,25]. This striking disparity suggests that the superior WSSV resistance of *M. nipponense* likely stems not from a more intense immune activation, but from a more efficient and targeted regulatory strategy that minimizes systemic disruption while maintaining essential cellular functions.

A key finding was the constitutive up-regulation of genes associated with cellular stress management and structural integrity in surviving *M. nipponense*. The significant enrichment of pathways involved in signal transduction [26], cellular processes, genetic information processing, transport, and catabolism indicates a coordinated effort to reinforce cellular homeostasis. This “proactive homeostatic reinforcement” strategy likely enhances the ability of *M. nipponense* cells to resist virus-induced stress, maintain energy balance, and efficiently execute immune functions without triggering catastrophic systemic failure. This perspective is further supported by the up-regulation of heat shock proteins (HSP70, HSP21), actin, and myosin heavy chain, highlighting their roles in maintaining protein stability and preserving muscle function, which are crucial for survival under pathogen challenge.

In contrast, *L. vannamei* exhibited distinct responses correlated with survival outcomes. Moribund individuals displayed transcriptomic signatures of severe metabolic exhaustion and tissue damage, as evidenced by the enrichment of pathways such as viral myocarditis, hypertrophic cardiomyopathy, and diabetic cardiomyopathy in gills, alongside metabolic pathways and chemical carcinogenesis in the hepatopancreas. This model is consistent with the study of Chen et al. in the hepatopancreas of *L. vannamei* infected with WSSV [4]. The down-regulation of key structural proteins (e.g., cuticle proteins, actin) and immune factors (e.g., GPI inositol deacylase, C-type lectins) reflected a state of systemic collapse. According to reports, at different stages of WSSV attack, the transcriptional expression of some stratum corneum proteins is significantly up-regulated or down-regulated, indicating that these proteins may be involved in the WSSV infection process [27,28,29]. C-type lectins are known for agglutination activity and play crucial roles in regulating the prophenoloxidase (*proPO*) activation system, enhancing phagocytosis and encapsulation, synthesizing antimicrobial peptides, and mediating antiviral immune responses [30].

Conversely, surviving *L. vannamei* mounted a more specific immune response, characterized by the enrichment of Toll-like receptor signaling, NOD-like receptor signaling, and apoptosis pathways. The innate immune response serves as the first line of defense against microbial infections in both insects and mammals [31]. It triggers a variety of humoral and cellular activities through signal transduction pathways that are conserved across both insects and mammals. Cellular immune responses include encapsulation, phagocytosis, coagulation, and melanization carried out by various hemocytes. Humoral immune responses involve the recognition of microorganisms, signal transduction—encompassing the Toll, IMD, and JAK/STAT pathways—and the production of immune effectors in the fat body [32]. The Toll-like receptor (TLR) signaling pathway and NOD-like receptors stand at the forefront of the recognition of extracellular and intracellular pathogens. They identify the most conserved structures of microorganisms and initiate responses to infection. These pathways play a crucial role in the innate immune system, conferring resistance against viral infections in a wide range of vertebrates and invertebrates [33,34,35]. This suggests that survival in *L. vannamei* may depend on the timely activation of specific innate immune pathways to control viral replication and infected cell death, although this response appears to be less effective than the homeostasis-focused strategy observed in *M. nipponense*.

Tissue-specific responses also provided valuable insights. The hepatopancreas and gill play a relevant role in the immune defense of crustaceans by producing immune proteins such as hemocyanin and lectins [36,37,38]. In *L. vannamei*, the gills and hepatopancreas served as hotspots of transcriptional activity following WSSV infection, reflecting their roles as primary sites for viral entry and immune response. Furthermore, among the tissues most severely affected by WSSV are mesodermal tissues, such as muscle [38]. In contrast, the muscle tissue of *M. nipponens* displayed a substantial number of DEGs, predominantly down-regulated, including genes related to cuticle and chitin metabolism. This may indicate a strategic reallocation of resources from exoskeletal maintenance toward internal defense and repair mechanisms during infection, a response not prominently observed in *L. vannamei*.

*Hsp70kDa* is one of the most prominent proteins studied across diverse organisms. It not only facilitates proper protein folding and prevents aberrant protein aggregation but also plays significant roles in immune responses and cell death [39,40]. In aquatic animals, exposure to stressors such as toxicants, infections, and oxidative stress induces the expression of *HSP* genes [41]. The rapid up-regulation of HSP genes represents a protective mechanism that supports cellular biochemical processes and helps mitigate physiological and histological alterations under adverse conditions [42].

The qRT-PCR results of ten randomly selected DEGs strongly validated the reliability of our transcriptomic data, confirming the accuracy of our sequencing and analytical pipeline. Furthermore, functional enrichment analyses (GO and KEGG) consistently highlighted fundamental differences in biological processes and pathways between the two species, further corroborating that distinct molecular strategies underlie their differential susceptibility to WSSV. Meanwhile, functional validation of the candidate gene HSP70kDa via RNA interference demonstrated that its knockdown led to a significant increase in both cumulative mortality and viral copy number in WSSV-infected *M. nipponense*.

In summary, our study demonstrates that the high resistance of *M. nipponense* to WSSV is associated with a targeted transcriptomic response aimed at maintaining cellular integrity and homeostasis. In contrast, the response in *L. vannamei* is more extensive and variable between lethal and survival outcomes, with survival depending on the activation of specific immune pathways. These findings not only deepen our understanding of the molecular basis of WSSV resistance in crustaceans but also identify potential key genes and pathways (e.g., *HSPs*, cytoskeletal proteins, core cellular maintenance pathways) that could be targeted for future genetic improvement or therapeutic strategies in susceptible shrimp species, such as *L. vannamei*.

## 4. Materials and Methods

### 4.1. Experimental Animals

Healthy specimens of *M. nipponense* and *L. vannamei* were obtained from the Freshwater Fisheries Research Center, Chinese Academy of Fishery Sciences (Wuxi, Jiangsu Province, China), which were maintained as specific pathogen-free (SPF) stocks. Prior to the experiment, all prawns were acclimated for seven days in a laboratory recirculating aquaculture system. During acclimation, water temperature was maintained at 25 ± 1 °C, pH at 7.5–8.0, with continuous aeration. The prawns were fed with *Paludina* spp. twice daily (morning and evening) at a ration of approximately 3–5% of their total body weight, and the culture water was refreshed daily. Sex was not considered as a variable in this study. For each species, both control and WSSV-challenged (experimental) groups were established, with three biological replicates per group and fifteen prawns per replicate.

The WSSV strain was isolated from diseased *L. vannamei*, provided by the School of Life Sciences, Sun Yat-sen University, and confirmed via PCR identification and sequencing verification. The viral inoculum was prepared following an established protocol in our laboratory [3]. Briefly, muscle tissue from shrimp with severe WSSV infection was homogenized, centrifuged, and the supernatant was filtered through a 0.40 μm membrane for sterilization. The copy number of the viral stock was quantified using absolute quantitative PCR and aliquots were stored at −80 °C for subsequent use.

Prior to the formal infection experiment, the challenge dose was determined based on the median lethal dose (LD_50_) established in our previous research [3]. The LD_50_ for *L. vannamei* is 10^0.59^ copies/g, while for *M. nipponense*, it is 10^3.84^ copies/g. To ensure a comparable infection pressure and to observe differential responses, this study applied the same challenge dose, corresponding to the LD_50_ for the susceptible species *L. vannamei* (10^0.59^ copies/g), to both species. For the challenge, the viral stock was diluted to the required concentration with sterile PBS, and each prawn was injected via the pericardial cavity with 2 μL of the suspension. The control groups received an equivalent volume of sterile PBS.

At 24 h post-injection (hpi), prawns were closely observed and categorized based on their clinical status. In *L. vannamei*, individuals displaying significantly reduced swimming activity, loss of balance, or consistent sideways swimming were classified as moribund, while those showing no discernible behavioral differences compared to the uninfected controls were classified as survivors. In contrast, no moribund individuals were observed in *M. nipponense* at this time point; all challenged prawns appeared behaviorally normal and were therefore classified as survivors. Subsequently, tissues including the hepatopancreas, gills, and muscle were collected from three biological replicates per group: Control and Surviving groups for both species, plus an additional Moribund group for *L. vannamei*. All collected samples were immediately flash-frozen in liquid nitrogen and stored at −80 °C for subsequent RNA extraction.

### 4.2. cDNA Library Construction and Sequencing

Total RNA was extracted from the gills, hepatopancreas, and muscle tissues of *L. vannamei* and *M. nipponense* using the Trizol reagent (Life Technologies, Carlsbad, CA, USA) according to the manufacturer’s protocol. Briefly, tissues were fully ground in liquid nitrogen, homogenized in Trizol, and subjected to phase separation with chloroform. RNA was precipitated from the aqueous phase with isopropanol, washed with ethanol, and dissolved in RNase-Free water.

The quantity and quality of the extracted total RNA were assessed using an Agilent 2100 Bioanalyzer (Agilent Technologies, Santa Clara, CA, USA) and a NanoDrop ND-2000 spectrophotometer (Thermo Fisher Scientific, Wilmington, DE, USA). Only high-quality RNA samples were used for subsequent library construction; all samples met the criteria of OD260/280 ratio of 1.8–2.0, OD260/230 ratio of 2.0–2.2, and an RNA Integrity Number (RIN) greater than 7.0.

Strand-specific RNA-seq libraries were constructed using the Hieff NGS^®^ Ultima Dual-mode mRNA Library Prep Kit (Yeasen Biotechnology Co., Ltd., Shanghai, China) following the manufacturer’s instructions. The process included poly(A)+ mRNA enrichment using oligo(dT) magnetic beads, RNA fragmentation, first- and second-strand cDNA synthesis, end repair, A-tailing, adapter ligation, and PCR amplification. Library quality and size distribution were verified using the Agilent 2100 Bioanalyzer with the High Sensitivity DNA assay kit (Agilent Technologies, Santa Clara, CA, USA). Finally, the prepared libraries were sequenced on an Illumina HiSeq X Ten platform at Genedenovo Biotechnology Co., Ltd. (Guangzhou, China).

### 4.3. RNA-Seq Data Analysis

Raw sequencing data were processed with fastp (version 0.23.2) to ensure data quality. The filtering steps included removal of reads containing adapter sequences, reads with more than 10% unknown bases (N), and low-quality reads (where over 50% of the bases had a quality score ≤ 20). The resulting high-quality reads were then aligned to the reference genome of *M. nipponense* (NCBI Assembly Accession: GCA_015104395.1, https://ftp.ncbi.nlm.nih.gov/genomes/all/GCF/015/104/395/GCF_015104395.2_ASM1510439v2/) (accessed on 15 October 2024) and the reference genome of *L. vannamei* (NCBI Assembly Accession: GCA_003789085.1, https://www.ncbi.nlm.nih.gov/datasets/genome/GCF_003789085.1/) (accessed on 15 October 2024) using HISAT2 (version 2.2.1).

Gene expression levels were quantified as Fragments Per Kilobase of transcript per Million mapped reads (FPKM) using RSEM (version 1.3.3). FPKM normalization accounts for both gene length and sequencing depth variations across samples. Differential expression analysis was performed with the DESeq2 (version 1.38.3) R package, which employs a negative binomial distribution model to account for over-dispersion in count data across biological replicates. The Wald test within DESeq2 was used to assess statistical significance. Genes with an absolute log2 fold change (|log2FC|) > 1 (corresponding to a fold change > 2) and a false discovery rate (FDR) adjusted *p*-value < 0.05 were considered differentially expressed genes (DEGs). The use of FDR correction is a standard approach to control for multiple testing in transcriptomic studies, balancing the control of false positives with statistical power.

### 4.4. Enrichment Analysis of DEGs

Functional enrichment analysis, including Gene Ontology (GO) (http://www.geneontology.org) (accessed on 30 October 2024), Kyoto Encyclopedia of Genes and Genomes (KEGG) (http://www.genome.jp/kegg/) (accessed on 30 October 2024) pathway analyses, was conducted separately for the DEGs from each comparison set using the clusterProfiler (version 4.6.2) R package with the standard GO and KEGG databases. Terms and pathways with an E-value ≤ 1 × 10^−5^ were considered significantly enriched.

The analysis covered the following comparison sets: for *M. nipponense*, three comparison sets (M-Co-Gi vs. M-Su-Gi, M-Co-He vs. M-Su-He, M-Co-Mu vs. M-Su-Mu) and for *L. vannamei*, six comparison sets (L-Co-Gi vs. L-Su-Gi, L-Co-Gi vs. L-Mo-Gi, L-Co-He vs. L-Su-He, L-Co-He vs. L-Mo-He, L-Co-Mu vs. L-Su-Mu, L-Co-Mu vs. L-Mo-Mu).

### 4.5. Statistical Analysis Validation by Quantitative PCR and Related Analysis

Differentially expressed genes (DEGs) from the three comparison sets in *M. nipponense* and the six comparison sets in *L. vannamei* were ranked by absolute fold change. The top 20 up-regulated and top 20 down-regulated DEGs from each set were listed, which is provided in Supplementary Table S3. Ten DEGs were selected for validation based on the most extreme log2 fold changes in specific comparison sets, representing the strongest transcriptional responses. Specifically, we selected the most down-regulated genes from M-Co-Gi-vs-M-Su-Gi, L-Co-He-vs-L-Su-He, and L-Co-Mu-vs-L-Mo-Mu; the most up-regulated genes from L-Co-Gi-vs-L-Mo-Gi, L-Co-Gi-vs-L-Su-Gi, and L-Co-He-vs-L-Mo-He; and the genes with the largest absolute fold change (regardless of direction) from M-Co-He-vs-M-Su-He and M-Co-Mu-vs-M-Su-Mu. A total of 10 genes were randomly selected for experimental validation by quantitative PCR (qPCR). This random selection from significantly altered transcripts aimed to provide unbiased verification of the transcriptome sequencing accuracy.

Gene-specific primers were designed using NCBI Primer-BLAST (https://www.ncbi.nlm.nih.gov/tools/primer-blast/, accessed on 16 July 2025) to span exon–exon junctions where possible, ensuring specificity. Primer sequences are listed in Supplementary Table S4. The qPCR reactions were performed using Taq SYBR Green qPCR Premix (Universal) (Jiangsu Yugong Biotechnology Co., Ltd., Lianyungang, China) on a QuantStudio 6 Flex Real-Time PCR System (Applied Biosystems, Foster City, CA, USA). Each 20 μL reaction mixture contained 10 μL of Premix, 0.4 μL of each primer (10 μM), 1 μL of cDNA template, and 8.2 μL of RNase-free water. The thermal cycling protocol was: 95 °C for 30 s; followed by 35 cycles of 95 °C for 10 s and 60 °C for 30 s. A melt curve analysis was performed using the instrument’s default acquisition program at the end of each run to confirm primer specificity and the absence of primer dimers. The amplification efficiency (E) for each primer pair, calculated from standard curves using serial dilutions of pooled cDNA, ranged between 90% and 110%, and the correlation coefficient (R^2^) was >0.99.

For validation, cDNA samples derived from the same RNA used for RNA-seq were analyzed. This included three independent biological replicates per tissue per group. Each qPCR reaction was performed in triplicate (technical replicates). In the qPCR analysis, Eukaryotic Translation Initiation Factor (EIF) [43] and β-actin [4] genes were used as internal references for *M. nipponense* and *L. vannamei*, respectively, based on our previous stability assessments. The sequence of all primers is listed in Supplementary Table S4. The comparative Ct (2^−ΔΔCT^) method was used to calculate the relative fold change in gene expression. Data normalization was first performed against the geometric mean of the reference gene (ΔCT), followed by comparison to the designated control group (ΔΔCT).

To compare expression levels between the control and treatment groups for each specific gene and tissue combination, which constitutes a two-group comparison (infected vs. control) within a defined context, a two-tailed Student’s *t*-test was applied. This approach is statistically appropriate for assessing the effect of the single primary factor (WSSV infection status) in each of these focused comparisons. All statistical analyses were conducted using SPSS 23.0 software. Data are presented as mean ± standard deviation from three independent biological replicates, and a *p*-value < 0.05 was considered statistically significant.

### 4.6. Screening of Common Candidate Genes for WSSV Resistance

To systematically identify conserved candidate genes associated with WSSV resistance, a comparative analysis was performed based on KEGG pathway annotation, following these formal steps:

1. Selection of Top-responding DEGs: For each of the nine comparison sets (three in *M. nipponense* and six in *L. vannamei*), differentially expressed genes (DEGs) were ranked by the absolute value of their log2 fold change (|log2FC|). From each ranked list, the top 20 up-regulated and the top 20 down-regulated DEGs were extracted, representing the most robust transcriptional responses in each specific experimental context.
2. Cross-species Comparison via KEGG Orthology: The KEGG Orthology (KO) identifiers corresponding to these selected top DEGs were compiled. We then conducted a pairwise comparison across species to identify KO identifiers that met two criteria: (i) present in the top DEG lists of both *M. nipponense* and *L. vannamei*, and (ii) associated with DEGs exhibiting a consistent direction of regulation (i.e., both up-regulated or both down-regulated in response to WSSV) [44].
3. Candidate Gene Identification: Genes annotated with the shared and consistently regulated KO identifiers were prioritized as high-confidence common candidate genes implicated in WSSV resistance. Through this analysis, heat shock protein 70 kDa (*HSP70*, KEGG Orthology ID: K03283) was identified as a prominent candidate due to its strong up-regulation in both species.

The complete list of the top 20 up- and down-regulated DEGs for each comparison set, including gene identifier, log2FC, adjusted *p*-value, and KEGG annotation, is provided in Supplementary Table S3.

### 4.7. RNA Interference Experiment

Healthy *M. nipponense* prawns were obtained from the same population as the primary WSSV challenge experiment (Freshwater Fisheries Research Center, Chinese Academy of Fishery Sciences, Wuxi, China). The average body weight was 0.82 ± 0.30 g. A total of 180 prawns were acclimated and randomly divided into two groups: (1) dsMn-HSP70 experimental group and (2) dsMn-GFP control group. Each group consisted of three biological replicates, with 30 prawns per replicate. Prawns received a single intramuscular injection into the pericardial cavity with dsRNA at a dose of 4 μg per gram of body weight in a volume of 2 μL. On days 1, 4, and 7 post-injection, 3 prawns were randomly sampled from each replicate (9 prawns per group per time point) for muscle tissue collection. Knockdown efficiency was assessed by qRT-PCR using the same protocol and reference gene (EIF) as described in Section 4.5. The time point at which the lowest expression level of the target gene was achieved was determined for subsequent functional analyses.

The full-length cDNA sequence of the candidate gene *Mn-HSP70* (Heat Shock Protein 70 kDa, Genbank accession No.: PX754291. KEGG ID: K03283) was obtained from the *M. nipponense* transcriptome. A specific fragment of *Mn-HSP70* was selected for dsRNA synthesis. The green fluorescent protein (GFP) gene was used as a non-target control. Gene-specific primers, incorporating a T7 promoter sequence, were designed using the SnapDragon—dsRNA Design online tool (https://www.flyrnai.org/snapdragon, accessed on 16 July 2025). Primer (*Mn-HSP70*) sequences are listed in Supplementary Table S4.

### 4.8. Experimental Challenge of dsRNA-Injected M. nipponense with WSSV

Healthy *M. nipponense* prawns were obtained from the same population as the primary WSSV challenge experiment (Freshwater Fisheries Research Center, Chinese Academy of Fishery Sciences, Wuxi, China). The average body weight was 0.54 ± 0.10 g. A total of 540 *M. nipponense* specimens were acclimatized for seven days and randomly divided allocated into two identical cohorts (n = 270 each), for viral load quantification and mortality recording, respectively. Each cohort was further divided into three experimental groups (with three replicates per group, n = 30 per replicate): (1) dsMn-HSP70 kDa + WSSV (experimental group), (2) dsMn-GFP + WSSV (positive control), and (3) dsMn-GFP + PBS (dsRNA control). Individuals received an intramuscular injection into the pericardial cavity of either dsMn-HSP70 kDa or dsMn-GFP At the time point of peak RNA interference efficiency, shrimp were subsequently challenged via injection with either WSSV viral suspension or PBS (control). The challenge dose was standardized to 10^3.84^ copies per gram of body weight, which corresponds to the previously determined median lethal dose (LD_50_) for *M. nipponense* [3], with an injection volume of 2 µL.

Viral Copy Number Determination (n = 270 prawns): Muscle tissue (~500 mg per prawn) was collected from 3 prawns per biological replicate at each time point (24, 48, 72, 96, 120, 144 hpi). This resulted in 9 independent muscle samples per group per time point (3 biological replicates × 3 prawns each). Absolute qPCR for WSSV Quantification: Total DNA was extracted from approximately 25 mg of muscle tissue using the TaKaRa MiniBEST Universal Genomic DNA Extraction Kit Ver.5.0 (Takara Bio, Kusatsu, Japan), following the manufacturer’s protocol. The concentration and purity of extracted DNA were measured with a NanoDrop spectrophotometer (Thermo Fisher Scientific, Wilmington, DE, USA). Reactions were performed using the same Taq SYBR Green qPCR Premix (Universal) and on the same QuantStudio 6 Flex system (Thermo Fisher Scientific, Waltham, MA, USA) as described for gene expression analysis (Section 4.5). WSSV copy number was determined by absolute qPCR targeting the viral vp28 gene. The quantitative standard was based on a recombinant plasmid containing the vp28 gene fragment. The standard curve showed a slope of -3.53 and efficiency of 92%, with an R^2^ > 0.99, constructed and validated in our laboratory.

Cumulative Mortality Recording (n = 270 prawns): Cumulative mortality was recorded at 12 h intervals until 144 h after WSSV challenge.

Statistical significance analysis was then performed on viral copy numbers and cumulative mortality between the experimental group (dsMn-HSP70kDa + WSSV) and each of the two control groups independently: dsMn-GFP + WSSV (positive control) and dsMn-GFP + PBS (dsRNA control). No comparisons were conducted between the two control groups.

## 5. Conclusions

This study represents the first investigation into the molecular mechanism underlying WSSV resistance in *M. nipponense*, employing *L. vannamei* as a comparative model. This comparative transcriptomic study revealed that the robust WSSV resistance in *M. nipponense* stems from a “proactive homeostatic reinforcement” strategy, characterized by the up-regulation of heat shock proteins and structural genes, and the enrichment of cellular process and transport pathways, thereby fortifying cellular homeostasis without massive immune activation. In contrast, the survival of *L. vannamei* relied on the activation of specific immune pathways such as Toll-like receptor signaling. Additionally, functional validation was conducted using RNA interference (RNAi), which identified HSP70 as a critical gene conferring antiviral resistance. These findings elucidate distinct molecular mechanisms of WSSV defense in crustaceans and provide crucial genetic targets for breeding disease-resistant shrimp.

## Data Availability

All sequence reads were deposited in NCBI (accession SRX31176670-SRX31176714) under Bioproject PRJNA1367779. The original contributions presented in this study are included in the article/Supplementary Materials. Further inquiries can be directed to the corresponding authors.

## Supplementary Materials

The supporting information can be downloaded at: https://www.mdpi.com/article/10.3390/ijms27020766/s1.

## References

[1] Cabansag Y.C., Lazaro J.V., Yambot A.P. (2014). Quantitative Gene Expression of C-Type Lectin in *Litopenaeus vannamei* Collected from Shrimp Farms with *White Spot Syndrome Virus* Disease Outbreak. Philipp. Agric. Sci..

[2] Lightner D.V. (2011). Virus Diseases of Farmed Shrimp in the Western Hemisphere (the Americas): A Review. J. Invertebr. Pathol..

[3] Zhao C.Y., Fu H.T., Sun S.M., Qiao H., Zhang W.Y., Jin S.B., Jiang S.F., Xiong Y.W., Gong Y.S. (2017). Experimental Inoculation of Oriental River Prawn *Macrobrachium nipponense* with White Spot Syndrome Virus (WSSV). Dis. Aquat. Org..

[4] Chen X.H., Zeng D.G., Chen X.L., Xie D.X., Zhao Y.Z., Yang C.L., Li Y.M., Ma N., Li M., Yang Q. (2013). Transcriptome Analysis of *Litopenaeus vannamei* in Response to White Spot Syndrome Virus Infection. PLoS ONE.

[5] Zhou J.F., Fang W.H., Yang X.L., Zhou S., Hu L.L., Li X.C., Qi X.Y., Su H., Xie L.Y. (2012). A Nonluminescent and Highly Virulent *Vibrio harveyi* Strain Is Associated with “Bacterial White Tail Disease” of *Litopenaeus vannamei* Shrimp. PLoS ONE.

[6] t Hoen P.A.C., Ariyurek Y., Thygesen H.H., Vreugdenhil E., Vossen R.H.A.M., de Menezes R.X., Boer J.M., van Ommen G.-J.B., den Dunnen J.T. (2008). Deep Sequencing-Based Expression Analysis Shows Major Advances in Robustness, Resolution and Inter-Lab Portability over Five Microarray Platforms. Nucleic Acids Res..

[7] Wongteerasupaya C., Vickers J.E., Sriurairatana S., Nash G.L., Akarajamorn A., Boonsaeng V., Panyim S., Tassanakajon A., Withyachumnarnkul B., Flegel T.W. (1995). A Non-Occluded, Systemic Baculovirus That Occurs in Cells of Ectodermal and Mesodermal Origin and Causes High Mortality in the Black Tiger Prawn *Penaeus monodon*. Dis. Aquat. Org..

[8] Pradeep B., Rai P., Mohan S.A., Shekhar M.S., Karunasagar I. (2012). Biology, Host Range, Pathogenesis and Diagnosis of *White Spot Syndrome Virus*. Indian J. Virol..

[9] Oidtmann B., Stentiford G.D. (2011). White Spot Syndrome Virus (WSSV) Concentrations in Crustacean Tissues—A Review of Data Relevant to Assess the Risk Associated with Commodity Trade. Transbound. Emerg. Dis..

[10] Fu H.T., Jiang S.F., Xiong Y.W. (2012). Current Status and Prospects of Farming the Giant River Prawn (*Macrobrachium rosenbergii*) and the Oriental River Prawn (*Macrobrachium nipponense*) in China. Aquac. Res..

[11] Zhao C., Fu H.T., Sun S.M., Qiao H., Zhang W.Y., Jin S.B., Jiang S.F., Xiong Y.W., Gong Y.S. (2018). A Transcriptome Study on *Macrobrachium nipponense* Hepatopancreas Experimentally Challenged with White Spot Syndrome Virus (WSSV). PLoS ONE.

[12] Margulies M., Egholm M., Altman W.E., Attiya S., Bader J.S., Bemben L.A., Berka J., Braverman M.S., Chen Y.-J., Chen Z. (2005). Genome Sequencing in Microfabricated High-Density Picolitre Reactors. Nature.

[13] Liu L., Li Y., Li S., Hu N., He Y., Pong R., Lin D., Lu L., Law M. (2012). Comparison of Next-Generation Sequencing Systems. BioMed Res. Int..

[14] Santos C.A., Blanck D.V., de Freitas P.D. (2014). RNA-Seq as a Powerful Tool for Penaeid Shrimp Genetic Progress. Front. Genet..

[15] Rasal K.D., Nandanpawar P.C., Swain P., Badhe M.R., Sundaray J.K., Jayasankar P. (2016). MicroRNA in Aquaculture Fishes: A Way Forward with High-Throughput Sequencing and a Computational Approach. Rev. Fish Biol. Fish..

[16] Yáñez J.M., Newman S., Houston R.D. (2015). Genomics in Aquaculture to Better Understand Species Biology and Accelerate Genetic Progress. Front. Genet..

[17] Flegel T.W. (2012). Historic Emergence, Impact and Current Status of Shrimp Pathogens in Asia. J. Invertebr. Pathol..

[18] Arulmoorthy M.P., Anandajothi E., Vasudevan S., Suresh E. (2020). Major Viral Diseases in Culturable Penaeid Shrimps: A Review. Aquac. Int..

[19] Zhu Y., Ding Q., Yang F. (2007). Characterization of a Homologous-Region-Binding Protein from White Spot Syndrome Virus by Phage Display. Virus Res..

[20] Tsai M.F., Lo C.F., van Hulten M.C.W., Tzeng H.-F., Chou C.M., Huang C.J., Wang C.H., Lin J.Y., Vlak J.M., Kou G.H. (2000). Transcriptional Analysis of the Ribonucleotide Reductase Genes of Shrimp White Spot Syndrome Virus. Virology.

[21] Li Z., Lin Q., Chen J., Wu J.L., Lim T.K., Loh S.S., Tang X., Hew C.-L. (2007). Shotgun Identification of the Structural Proteome of Shrimp White Spot Syndrome Virus and iTRAQ Differentiation of Envelope and Nucleocapsid Subproteomes. Mol. Cell. Proteom..

[22] Han F., Xu J., Zhang X. (2007). Characterization of an Early Gene (Wsv477) from Shrimp White Spot Syndrome Virus (WSSV). Virus Genes.

[23] Liu W.J., Chang Y.S., Wang A.H.J., Kou G.H., Lo C.F. (2007). White Spot Syndrome Virus Annexes a Shrimp STAT To Enhance Expression of the Immediate-Early Gene Ie1. J. Virol..

[24] Liu W.J., Chang Y.S., Wang C.H., Kou G.H., Lo C.F. (2005). Microarray and RT-PCR Screening for White Spot Syndrome Virus Immediate-Early Genes in Cycloheximide-Treated Shrimp. Virology.

[25] Li F., Li M., Ke W., Ji Y., Bian X., Yan X. (2009). Identification of the Immediate-Early Genes of White Spot Syndrome Virus. Virology.

[26] Liu H., Chen R., Zhang Q., Peng H., Wang K. (2011). Differential Gene Expression Profile from Haematopoietic Tissue Stem Cells of Red Claw Crayfish, *Cherax quadricarinatus*, in Response to WSSV Infection. Dev. Comp. Immunol..

[27] Wang B., Li F., Luan W., Xie Y., Zhang C., Luo Z., Gui L., Yan H., Xiang J. (2008). Comparison of Gene Expression Profiles of *Fenneropenaeus chinensis* Challenged with WSSV and Vibrio. Mar. Biotechnol..

[28] Leu J.H., Chang C.C., Wu J.L., Hsu C.W., Hirono I., Aoki T., Juan H.F., Lo C.F., Kou G.-H., Huang H.C. (2007). Comparative Analysis of Differentially Expressed Genes in Normal and White Spot Syndrome Virus Infected Penaeus Monodon. BMC Genom..

[29] Yang F., Li S., Li F., Xiang J. (2018). A Cuticle Protein from the Pacific White Shrimp *Litopenaeus vannamei* Involved in WSSV Infection. Dev. Comp. Immunol..

[30] Xue Q., Yang B., Luo K., Luan S., Kong J., Fu Q., Cao J., Chen B., Dai P., Xing Q. (2024). Characterization and Expression Analysis of the C-Type Lectin Ladderlectin in *Litopenaeus vannamei* Post-WSSV Infection. Biology.

[31] Magnadóttir B. (2006). Innate Immunity of Fish (Overview). Fish Shellfish Immunol..

[32] Qin Z., Babu V.S., Wan Q., Zhou M., Liang R., Muhammad A., Zhao L., Li J., Lan J., Lin L. (2018). Transcriptome Analysis of Pacific White Shrimp (*Litopenaeus vannamei*) Challenged by *Vibrio parahaemolyticus* Reveals Unique Immune-Related Genes. Fish Shellfish Immunol..

[33] Chu Q., Xu T. (2020). MicroRNA Regulation of Toll-like Receptor, RIG-I-like Receptor and Nod-like Receptor Pathways in Teleost Fish. Rev. Aquac..

[34] Langefeld T., Mohamed W., Ghai R., Chakrabotty T. (2009). Toll-like Receptors and NOD-Like Receptors: Domain Architecture and Cellular Signalling. Target Pattern Recognition in Innate Immunity; Advances in Experimental Medicine and Biology.

[35] Arts J.A.J., Cornelissen F.H.J., Cijsouw T., Hermsen T., Savelkoul H.F.J., Stet R.J.M. (2007). Molecular Cloning and Expression of a Toll Receptor in the Giant Tiger Shrimp, *Penaeus monodon*. Fish Shellfish Immunol..

[36] Song K.-K., Li D.-F., Zhang M.-C., Yang H.-J., Ruan L.-W., Xu X. (2010). Cloning and Characterization of Three Novel WSSV Recognizing Lectins from Shrimp *Marsupenaeus japonicus*. Fish Shellfish Immunol..

[37] Rao R., Zhu Y.B., Alinejad T., Tiruvayipati S., Lin Thong K., Wang J., Bhassu S. (2015). RNA-Seq Analysis of *Macrobrachium rosenbergii* Hepatopancreas in Response to Vibrio Parahaemolyticus Infection. Gut Pathog..

[38] Santos C.A., Andrade S.C.S., Fernandes J.M.O., Freitas P.D. (2020). Shedding the Light on *Litopenaeus vannamei* Differential Muscle and Hepatopancreas Immune Responses in White Spot Syndrome Virus (WSSV) Exposure. Genes.

[39] Janewanthanakul S., Supungul P., Tang S., Tassanakajon A. (2020). Heat Shock Protein 70 from *Litopenaeus vannamei* (*Lv*HSP70) Is Involved in the Innate Immune Response against White Spot Syndrome Virus (WSSV) Infection. Dev. Comp. Immunol..

[40] Roberts R.J., Agius C., Saliba C., Bossier P., Sung Y.Y. (2010). Heat Shock Proteins (Chaperones) in Fish and Shellfish and Their Potential Role in Relation to Fish Health: A Review. J. Fish Dis..

[41] Zhou Y.J., Messmer M.N., Binder R.J. (2014). Establishment of Tumor-Associated Immunity Requires Interaction of Heat Shock Proteins with CD91. Cancer Immunol. Res..

[42] Yuan K., Yuan F.-H., He H.-H., Bi H.-T., Weng S.-P., He J.-G., Chen Y.-H. (2017). Heat Shock 70 kDa Protein Cognate 5 Involved in WSSV Toleration of *Litopenaeus vannamei*. Dev. Comp. Immunol..

[43] Hu Y., Fu H.T., Qiao H., Sun S.M., Zhang W.Y., Jin S.B., Jiang S.F., Gong Y.S., Xiong Y.W., Wu Y. (2018). Validation and Evaluation of Reference Genes for Quantitative Real-Time PCR in *Macrobrachium nipponense*. Int. J. Mol. Sci..

[44] Peruzza L., Thamizhvanan S., Vimal S., Kumar K.V., Shekhar M.S., Smith V.J., Hauton C., Vijayan K.K., Hameed A.S.S. (2020). A comparative synthesis of transcriptomic analyses reveals major differences between WSSV-susceptible *Litopenaeus vannamei* and WSSV-refractory *Macrobrachium rosenbergii*. Dev. Comp. Immunol..

