# Supplementary material for: A Distinct Defense Strategy: The Molecular Basis of WSSV Tolerance in Macrobrachium nipponense Revealed by Comparative Transcriptomics with Litopenaeus vannamei
Source: Int J Mol Sci. 2026 Jan 12;27(2):766. doi: 10.3390/ijms27020766 (PMC12840910; doi:10.3390/ijms27020766)
Supplement: Supplementary file 1 [file ijms-27-00766-s001.zip › Supplementary Table S1.pdf]

Supplementary Table S1. Summary of RNA-seq data and quality control metrics for all samples of *Macrobrachium nipponense*.

Sample Name	clean reads	clean data (bp)	GC(%)	Q20(%)	Q30(%)	Mapped reads	Mapping ratio(%)
M-Co-Gi-1	41586700	6162010010	40.54%	98.13%	94.75%	29303484	82.05%
M-Co-Gi-2	43396720	6436059752	40.81%	98.24%	94.84%	30659411	83.80%
M-Co-Gi-3	38284412	5691519043	39.87%	98.58%	95.70%	25628236	82.99%
M-Co-He-1	44521528	6557339027	46.69%	98.69%	95.90%	39727133	91.53%
M-Co-He-2	38589198	5741652139	46.78%	98.56%	95.57%	34313636	91.27%
M-Co-He-3	37428888	5583897545	45.27%	98.63%	95.74%	32124765	90.95%
M-Co-Mu-1	40798396	6017020180	47.85%	98.54%	95.55%	37008252	93.26%
M-Co-Mu-2	38079526	5635189465	47.79%	98.25%	94.75%	33829947	92.40%
M-Co-Mu-3	38601478	5731770470	48.95%	98.20%	94.69%	35148533	93.48%
M-Su-Gi-2	41713854	6156130050	41.19%	98.35%	95.21%	29633208	84.23%
M-Su-Gi-1	48120624	7156450734	40.15%	97.98%	94.23%	32126927	82.50%
M-Su-Gi-3	38371872	5686642343	40.95%	98.59%	95.73%	27038356	85.33%
M-Su-He-1	37824568	5624336430	45.28%	98.66%	95.89%	32407550	90.74%
M-Su-He-2	41213726	6079308577	45.84%	98.52%	95.53%	35628195	91.01%
M-Su-He-3	44720738	6635502635	46.37%	98.27%	94.87%	35064358	81.83%
M-Su-Mu-1	42757304	6374298025	48.54%	98.20%	94.64%	36975247	88.95%
M-Su-Mu-2	38036936	5645077358	48.92%	98.29%	94.81%	34704189	93.89%
M-Su-Mu-3	40591380	6019672216	48.85%	98.52%	95.46%	37088368	94.10%
总计/平均值	734637848	6051882000	45.04%	98.40%	95.21%	598409795	88.57%

The table details the sequencing throughput and quality statistics for each biological replicate. Samples are labeled according to their group: control (M-Co) and survival (M-Su); and tissue: gill (Gi), hepatopancreas (He), and muscle (Mu). The metrics include:

Clean reads: The number of high-quality sequences retained after raw data filtering.

Clean data (bp): The total base pairs of clean data.

GC content (%): The percentage of guanine and cytosine nucleotides in the clean sequences.

Q20 (%) / Q30 (%): The percentage of bases with a Phred quality score greater than 20 (indicating a base call accuracy of 99%) or 30 (base call accuracy of 99.9%).

Mapped reads: The number of clean reads that were successfully aligned to the reference genome of *M. nipponense.*

Mapping ratio (%): The percentage of clean reads that were successfully aligned (Mapped reads / Clean reads × 100%).
