# Supplementary material for: A Distinct Defense Strategy: The Molecular Basis of WSSV Tolerance in Macrobrachium nipponense Revealed by Comparative Transcriptomics with Litopenaeus vannamei
Source: Int J Mol Sci. 2026 Jan 12;27(2):766. doi: 10.3390/ijms27020766 (PMC12840910; doi:10.3390/ijms27020766)
Supplement: Supplementary file 1 [file ijms-27-00766-s001.zip › Supplementary Table S2.pdf]

Supplementary Table S2. Summary of RNA-seq data and quality control metrics for all samples of *L. vannamei*.

Sample name	(clean reads)	clean data (bp)	GC（%）	Q20 (%)	Q30(%)	Mapped reads	Mapping ratio（%）
L-Co-Mu-1	41018216	6096025129	50.69%	97.53%	92.82%	35840660	89.27%
L-Co-Mu-2	35848282	5300158374	51.94%	98.50%	95.35%	32000593	90.37%
L-Co-Mu-3	41422546	6105767943	51.49%	98.57%	95.68%	36908484	90.42%
L-Co-Gi-1	39120228	5772545127	42.94%	97.91%	93.72%	29868175	88.36%
L-Co-Gi-2	47913336	7128976723	41.35%	98.04%	94.54%	33389245	87.74%
L-Co-Gi-3	46735184	6895427225	42.33%	98.78%	96.35%	34339654	88.31%
L-Co-He-1	38090300	5647590992	49.03%	98.06%	94.06%	32621164	88.66%
L-Co-He-2	42543306	6342612741	47.97%	98.71%	96.19%	36618449	89.95%
L-Co-He-3	44685772	6611232509	47.90%	98.55%	95.52%	38501922	89.27%
L-Mo-Mu-1	42060622	6234291827	51.45%	98.19%	94.56%	37474428	90.39%
L-Mo-Mu-2	43364010	6418958606	51.32%	98.08%	94.53%	38398979	90.09%
L-Mo-Mu-3	36184440	5347316128	51.18%	98.19%	94.63%	32356536	90.90%
L-Mo-Gi-1	46063122	6809647955	42.65%	98.53%	95.44%	33472798	88.26%
L-Mo-Gi-2	46006760	6781608807	44.04%	98.03%	94.13%	34885999	87.83%
L-Mo-Gi-3	38978126	5737834772	43.23%	97.51%	92.96%	28390975	86.43%
L-Mo-He-1	42809682	6334820528	48.25%	98.72%	96.13%	36399394	88.23%
L-Mo-He-2	40698180	5995945951	47.87%	97.99%	93.99%	33735590	87.67%
L-Mo-He-3	46429958	6892333868	49.24%	98.08%	94.42%	39581699	87.86%
L-Su-Mu-1	40738248	6052260359	51.16%	97.37%	92.49%	35535032	88.58%
L-Su-Mu-2	44894284	6644419857	51.51%	97.67%	93.32%	39589574	89.35%
L-Su-Mu-3	36456366	5375059574	51.53%	98.54%	95.51%	32316284	89.76%
L-Su-Gi-1	41236118	6092726452	43.37%	98.02%	94.12%	31639773	88.59%
L-Su-Gi-2	45122466	6704716790	42.34%	97.68%	93.45%	33108745	87.50%
L-Su-Gi-3	47302888	7019543925	42.61%	97.66%	93.38%	34425242	87.46%
L-Su-He-1	44851418	6666758984	47.65%	98.65%	95.97%	37588627	89.24%
L-Su-He-2	44897012	6694134487	47.42%	98.63%	95.90%	36894428	89.29%
L-Su-He-3	43366358	6394950138	48.74%	97.74%	93.48%	36984935	88.64%
总计/平均值	42549526.96	6299913547	47.45%	98.15%	94.54%	942867384	88.83%

The table details the sequencing throughput and quality statistics for each biological replicate. Samples are labeled according to their group: control (L-Co), moribund(L-Mo) and survival (L-Su); and tissue: gill (Gi), hepatopancreas (He), and muscle (Mu). The metrics include:

Clean reads: The number of high-quality sequences retained after raw data filtering.

Clean data (bp): The total base pairs of clean data.

GC content (%): The percentage of guanine and cytosine nucleotides in the clean sequences.

Q20 (%) / Q30 (%): The percentage of bases with a Phred quality score greater than 20 (indicating a base call accuracy of 99%) or 30 (base call accuracy of 99.9%).

Mapped reads: The number of clean reads that were successfully aligned to the reference genome of *L. vannamei*..

Mapping ratio (%): The percentage of clean reads that were successfully aligned (Mapped reads / Clean reads × 100%).
